# Characterization of morphological and biological aspects of venomous caterpillars of the genus *Lonomia* Walker (Lepidoptera: Saturniidae) in Colombia

**DOI:** 10.1371/journal.pone.0285010

**Published:** 2023-05-31

**Authors:** Diana M. Toro-Vargas, Camila González, Rodolphe Rougerie, Angela R. Amarillo-Suárez

**Affiliations:** 1 Centro de Investigaciones en Microbiología y Parasitología Tropical (CIMPAT), Departamento de Ciencias Biológicas, Universidad de los Andes, Bogotá D.C., Colombia; 2 Institut de Systématique, Évolution, Biodiversité (ISYEB), Muséum National d’Histoire Naturelle, CNRS, EPHE, Sorbonne Université, Université des Antilles, Paris, France; 3 Departamento de Ecología y Territorio, Pontificia Universidad Javeriana, Bogotá D.C., Colombia; Laboratoire de Biologie du Développement de Villefranche-sur-Mer, FRANCE

## Abstract

The genus *Lonomia* Walker, 1855 (Lepidoptera: Saturniidae) is of particular interest to the medical community, since the scoli of these caterpillars harbor a venom that induces hemorrhaging in humans. In Colombia, deadly encounters with *Lonomia achelous* (Cramer, 1777), have been reported since 2000. There is little information on the main biological and ecological aspects of this genus to help better understand and develop prevention strategies. This study aimed to describe morphological and biological aspects (especially of immature stages) of four recently reported species of *Lonomia* in Colombia that pose a risk to humans. We collected caterpillars and adults from five localities and reared them under laboratory conditions. Specimens were identified using DNA barcoding and dissection of adult male genitalia. We provided the first description, to our knowledge, of part of the life cycles of *Lonomia casanarensis* Brechlin, 2017 and *Lonomia orientoandensis* Brechlin & Meister, 2011 and the complete life cycles of *Lonomia columbiana* Lemaire, 1972 and *Lonomia orientocordillera* Brechlin, Käch & Meister, 2013. We also present the first records of the parasitoids of *L*. *orientocordillera*, and *L*. *casanarensis* and new host plants. This information will guide not only their morphological recognition and the identification of their parasitoids and hosts, but also will guide rearing methods of these and other *Lonomia* species in new studies to prevent incidents with humans and create specific antivenoms.

## Introduction

The Lepidopteran genus *Lonomia* (Saturniidae) Walker, 1855 is distributed from Mexico to Argentina and comprises 60 species of medium-sized saturniids [1]. Their wings, viewed dorsally, range from yellow to orange and brown, sometimes with a reddish appearance. The forewings have a slightly convex costa with a right angle at the tip. The hindwings are broader than the forewings and have the same color. The forewings have one or sometimes two conspicuous white dots in the median area, sometimes surrounded by a black ring. The postmedial line of the fore- and hindwings are brown to black with a conspicuous to smooth white line on the inner part. The postmedial line of the forewings is straight, ending right before the apex of the wing. This single conspicuous darker band crossing the wings mimics a leaf vein. When adults naturally pose on a surface, they cover their hindwings with their forewings. Then, they can look like withered leaves. This pattern is very conserved and makes species identification very challenging and requiring the use of characters invisible to the naked eye, such as genitalia morphology, or the use of genetic markers such as DNA barcodes.

This genus is of medical importance due to the severity of the hemorrhagic syndrome that humans can experience after having contact with its larval stages. The prominent scoli of *Lonomia* caterpillars have spines with an internal channel in which a poisonous secretion is produced by a glandular cell located in its subapical region [2]. Contact with the spines provokes their fracture, and the venom is ejected as a defense mechanism [3]. In humans, this venom can cause a burning sensation, pain, and headaches [4], alter the coagulation system [5,6], and trigger other disorders, such as chronic or acute renal failure [7] and cerebral hemorrhage, causing death in some cases [8]. The current treatment for envenomation by *Lonomia* is administration of an antivenom produced against *Lonomia obliqua* Walker, 1855 by the Butantan Institute of Brazil [9], which has proven to be effective in response to envenomation by three species in Colombia: *L*. *descimoni* [10], *L*. *orientoandesis* [11] and *L*. *casanarensis* [11].

In Colombia, the number of accidents has increased since the first two cases were described in 2000 in the Casanare area, where the presence of *Lonomia* spp. is related to the increasing urbanization of rural areas [12]. The first accident was attributed to *L*. *achelous* [13], but given that the number of described species has increased from 11 [14] to 60 [1] in the last 20 years, it has been shown that the number of species involved in the accidents is wider [1].

Despite their diversity and medical relevance, there are very few studies describing the biology and ecology of *Lonomia* species [12,15–22]. Information regarding their species distribution, reproductive cycles, and interactions with host plants, predators and parasitoids is scarce. The best-known species is *L*. *obliqua*, because of its epidemiological importance in Brazil [16–20,22].

Improving our knowledge of the biological aspects of species with medical importance is critical to better design accident prevention strategies. For instance, knowing the duration of their life cycles can help to estimate seasonality throughout the year and, by identifying the duration of the larval stages, the seasons when accidents are more likely to occur. Additionally, the identification of natural parasitoids and host plants can provide valuable information for the management of integrated control strategies in areas under high land use transformation.

Although species identification can be performed based on the morphology of adult genitalia or the use of DNA barcodes, knowing the morphological differences in the larval stages across *Lonomia* species can facilitate preliminary identification in the field, especially when caterpillars can be examined in the urgency context of an envenomation accident. When reaching their sixth and last instar, *Lonomia* caterpillars are approximately 6 cm long and have a head with whitish frontal lines and brown bodies [20] covered with tree-shaped spiny scoli, which represent the typical armature of caterpillars in the Hemileucinae subfamily [14]. Wolfe and Balcázar-Lara [21] described a white H-shaped spot on the third thoracic segment of the fourth to sixth instar caterpillar of *Lonomia electra* Druce, 1886 that can be observed in other *Lonomia* species. Caterpillars are gregarious, living in large colonies of up to 200 individuals [14].

A recent taxonomic checklist for the Saturniidae of Colombia [23] recorded 13 *Lonomia* species: *L*. *columbiana*, *Lonomia descimoni* Lemaire, 1972, *Lonomia rufescens* Lemaire, 1972, *L*. *achelous*, *Lonomia venezuelensis* Lemaire, 1972, *Lonomia puntarenasiana* Brechlin & Meister, 2011, *L*. *orientoandensis*, *Lonomia madrediosiana* Brechlin & Meister, 2011, *L*. *orientocordillera*, *Lonomia laalbania* Brechlin, 2017, *Lonomia minca* Brechlin, 2017, *L*. *casanarensis* and *Lonomia rengifoi* Brechlin & Käch, 2017. However, a complementary study in which the authors participate [1] excluded *L*. *madrediosiana*, from the list of Colombian species and reported three additional species for Colombia: *Lonomia vanschaycki* Brechlin, Käch & Meister, 2013, *Lonomia cayennensis* Brechlin & Meister 2019, and an unidentified species, provisionally named as *Lonomia* CGR01. The aim of this study was to describe for the first time the morphological and biological aspects of four species of *Lonomia* collected in Colombia. We provide a detailed description of the life cycle of reared individuals collected from colonies in the field, larval and adult morphology, and information on the collection sites, including the parasitoids and the host plants.

## Results

We collected 48 adults of two species: *L*. *orientocordillera* (females n = 1, males n = 5) and *L*. *columbiana* (females n = 7, males n = 35). Females of these species laid eggs, allowing us to rear individuals for an entire life cycle from egg to adult. We also collected eight caterpillar colonies of *L*. *orientocordillera*, *L*. *orientoandensis* and *L*. *casanarensis* at different larval stages. We raised a total of 392 individuals (Table 1), from which 56 adults were obtained. Thus, we describe for the first time the complete life cycles of *L*. *columbiana* and *L*. *orientocordillera* and the partial life cycles of *L*. *casanarensis* and *L*. *orientoandensis*.

**Table 1:** Results of each evaluated trait by species.

Trait	Species			
	*Lonomia columbiana*	*Lonomia orientocordillera*	*Lonomia orientoandensis*	*Lonomia casanarensis*
Number of colonies	1	4	2	3
No. individuals reared	34	134	48	176
Egg height (mean mm ± std)	2.277 ± 0.052	2.335 ± 0.043	unknown	unknown
Greater egg diameter (mean mm ± std)	1.843 ± 0.064	1.834 ± 0,076	unknown	unknown
Smaller egg diameter (mean mm ± std)	1.679 ± 0.067	1.789 ± 0.057	unknown	unknown
Total larvae growth ratio	1.372	1.327	1.351	1.275
Egg to adult development Time (days)	116 to 146	145 to 214	unknown	unknown
Female pupa weight (mean g ± std)	3.156 ± 0.100	2.982 ± 0.399	2.528 ± 0.463	1.496 ± 0.264
Male pupa weight (mean g ± std)	1.084 ± 0.000	1.630 ± 0.368	1.884 ± 0.294	1.005 ± 0.155
Female pupa length (mean cm ± std)	4.186 ± 0.050	3.870 ± 0.500	3.772 ± 0.231	3.277 ± 0.188
Male pupa length (mean cm ± std)	2.927 ± 0.000	3.469 ± 0.179	3.119 ± 0.775	2.923 ± 0.122
Adult female body size (mean cm ± std)	2.944 ± 0.146	2.952 ± 0.339	2.581 ± 0.431	2.173 ± 0.237
Adult male body size (mean cm ± std)	2.314 ± 0.192	2.445 ± 0.179	1.919 ± 0.089	2.041 ± 0.049
Adult female wingspan (mean cm ± std)	9.200 ± 0.486	9.377 ± 0.272	8.130 ± 0.345	7.335 ± 0.397
Adult male wingspan (mean cm ± std)	7.351 ± 0.522	7.141 ± 0.568	6.749 ± 0.595	6.319 ± 0.412
Total egg to adult mortality (%)	84.09	69.73	85.79	77.57
Mortality caused by parasitoids (%)	0	33–38^a^	0	13^a^
Parasitoid species		*Lespesia sp*. (Robineau-Desvoidy) (Diptera: Tachinidae)		*Lespesia sp*.
		*Enicospilus* sp. (Stephens) (Hymenoptera: Ichneumonidae)		

^a^ The mortality in these two species was calculated from collected instars to adults in each colony.

**Fig 1:**
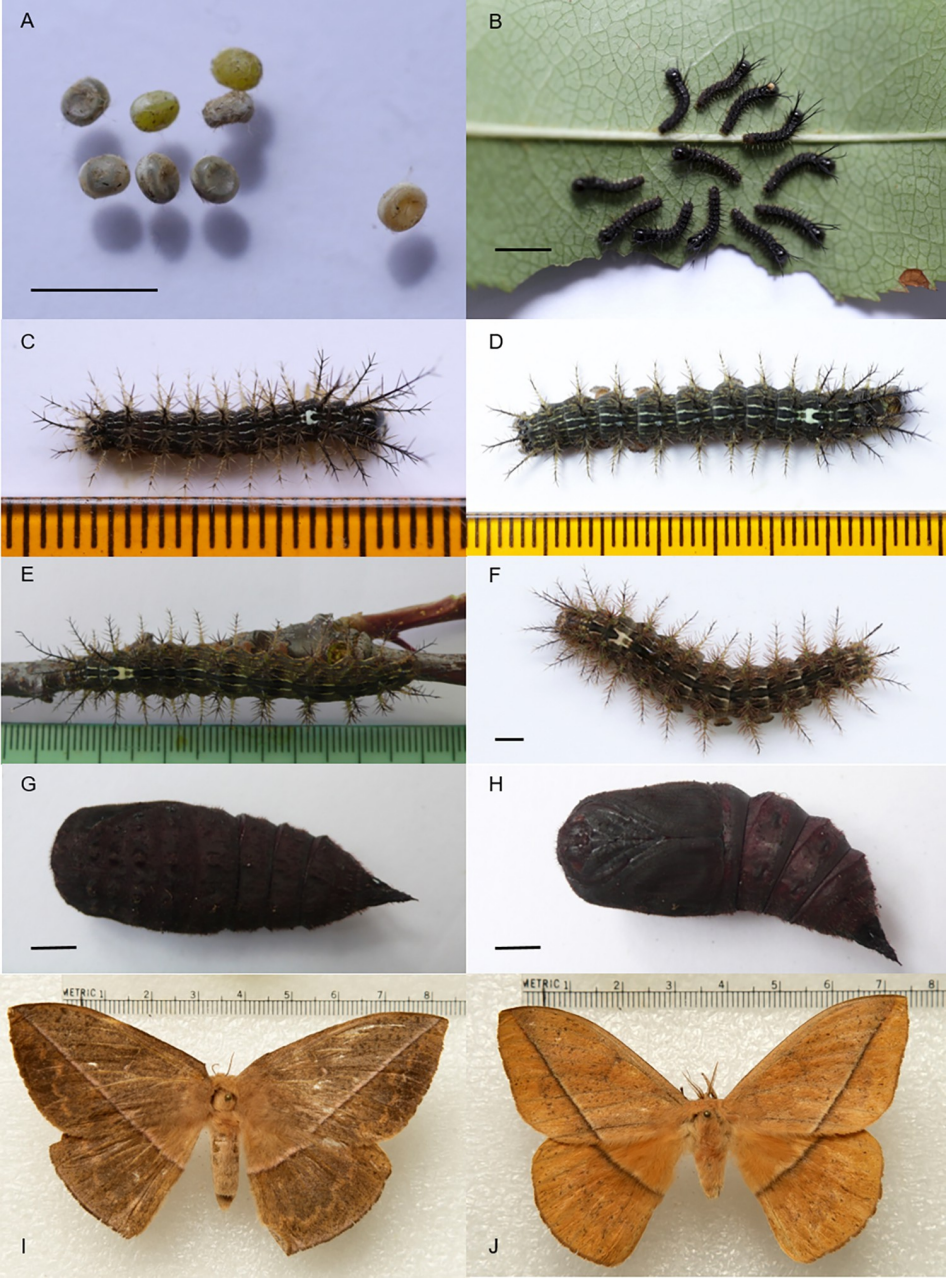
*Lonomia columbiana* life cycle. (A) Eggs. (B) First instar larvae. (C) Third instar larva. (D) Fourth instar larva. (E) Fifth instar larva. (F) Sixth instar larva. (G) Pupae dorsal view. (H) Pupae ventral view. (I) Female. (J) Male. Bar = 0.5 cm.

We found several features of immature stages that were shared by all species, including eggs that were oval and slightly flattened (Table 1), with a smooth surface and a light green color immediately after being oviposited (Fig 1A). Fertilized eggs are distinguishable from unfertilized eggs by their paler green appearance with a whitish band on the periphery as they approach hatching. The micropillar area was not evident in any of the eggs examined.

Caterpillars showed gregarious habits since the first instar (Fig 1B), resting during the day in places with little illumination, such as small branches of the feeding plant. In the field, individuals from colonies were found resting in the lower parts of the trunks of large trees. Individual caterpillars rested with their heads facing outwards from the center of the colony. Feeding occurred during the afternoon and at nighttime, when larvae ascended the tree to feed on leaves in the tree canopy. Caterpillars from the fourth to the sixth instar showed defensive behavior when touched, consisting of a rapid, “jumping” movement with a simultaneous raising of the head and rear end. It is worth noting that these are places where the largest spines are located. All caterpillar instars had the following characteristics: hypognathous head; stemmata 1, 2, 3, 4 and 6 forming a semicircle and 5 displaced ventrally; biordinal crochets with homoideous mesoseries; body segments with scoli forming bifid spines in first instar whose branches become denser as they grow; T1 (prothorax)-A7 (7^th^ abdominal segment) with one dorsal, subdorsal and lateral scoli on each side. A8-A9 with a single mid-dorsal scolus and one subdorsal and one lateral scolus on each side. A10 with a very reduced dorsal scolus. Subventral scoli are present in T1-A2 and A7-A10. The average growth ratio of larvae, that is the average cephalic capsule width of one instar divided by that of the previous caterpillar instar (for example, for *L*. *columbiana* 5.226 mm in the sixth instar was divided by 3.825 mm in the fifth instar), in all species was 1.3.

The prepupal stage was characterized by caterpillars that were no longer feeding nor remaining on the host plant and found at the bottom of the rearing box. There they look for a humid place in the soil to burrow into or rest to pupate. Then they retracted their body longitudinally, exhibiting a more robust appearance, especially in the first abdominal segments. Their color darkened, their displacement was short, and their defense reaction was a slow bending.

The cuticle of newly formed pupae hardened in one to four hours, acquiring a darker brown coloration as time passes. Female pupae are larger and heavier than males (Table 1).

**Fig 2:**
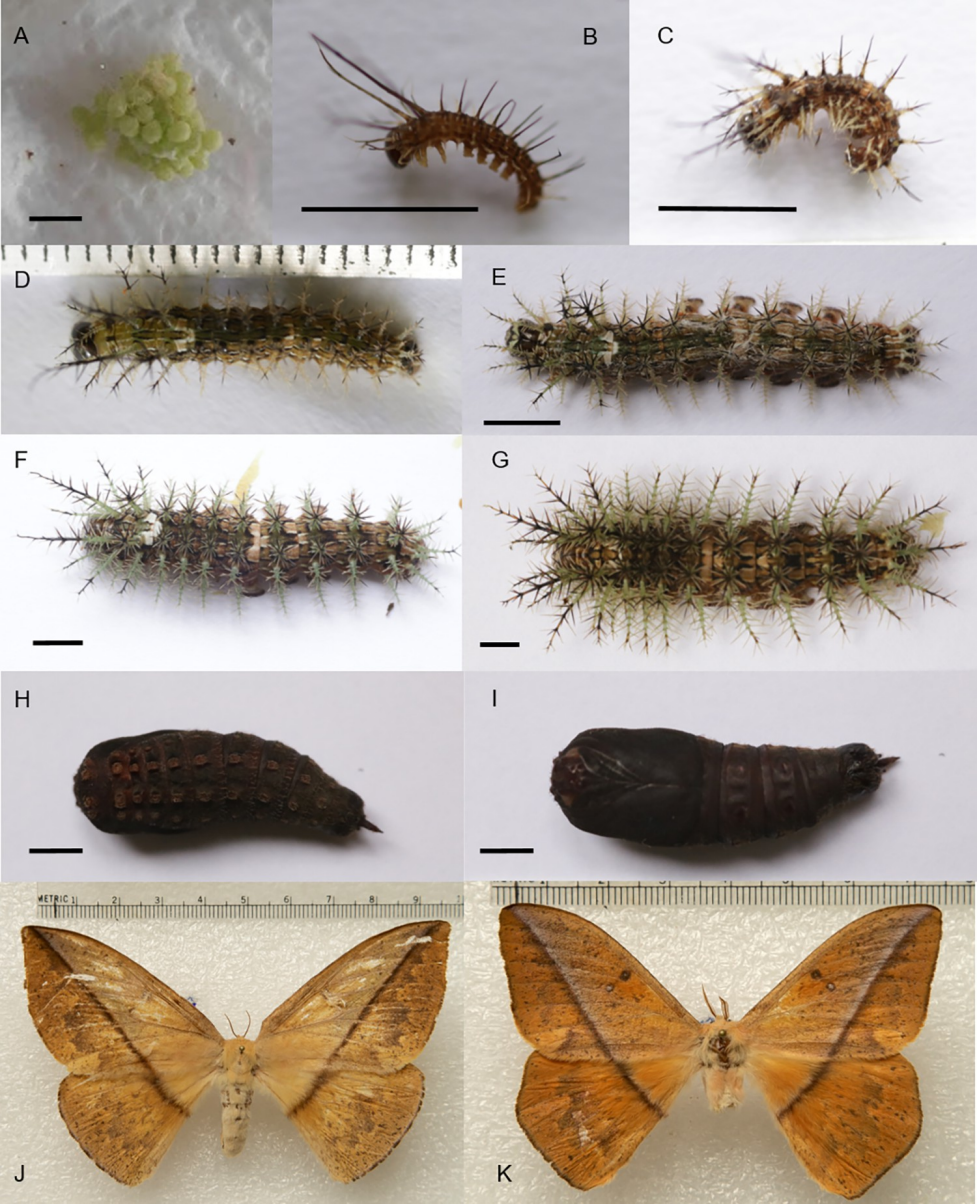
*Lonomia orientocordillera* life cycle. (A) Eggs. (B) First instar larvae. (C) Second instar. (D) Third instar larva. (E) Fourth instar larva. (F) Fifth instar larva. (G) Sixth instar larva. (H) Pupae dorsal view. (I) Pupae ventral view. (J) Female. (K) Male. Bar = 0.5 cm.

**Fig 3:**
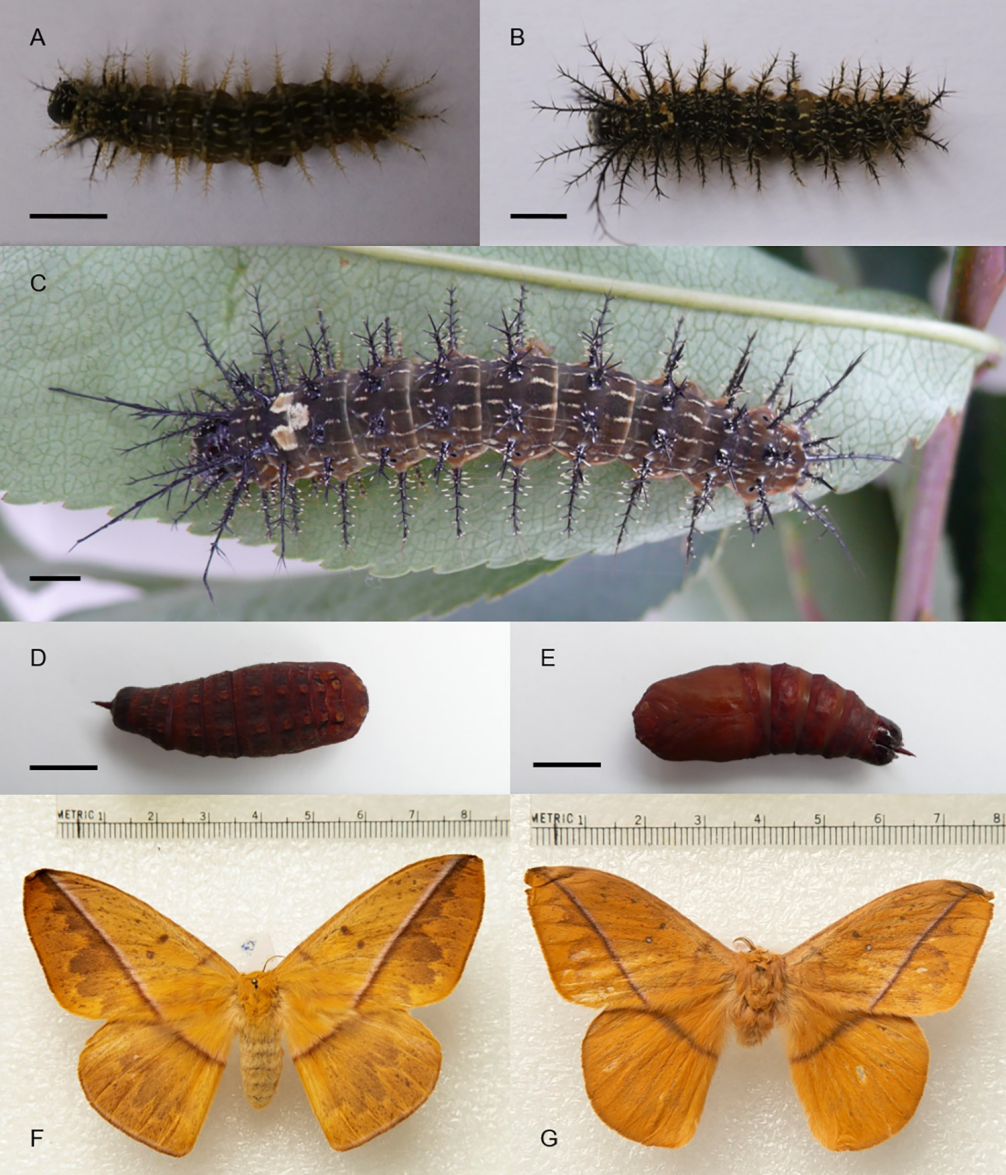
*Lonomia orientoandensis* life cycle. (A) Fourth instar larva. (B) Fifth instar larva. (C) Sixth instar larva. (D) Pupae dorsal view. (E) Pupae ventral view. (F) Female. (G) Male. Bar = 0.5 cm.

**Fig 4:**
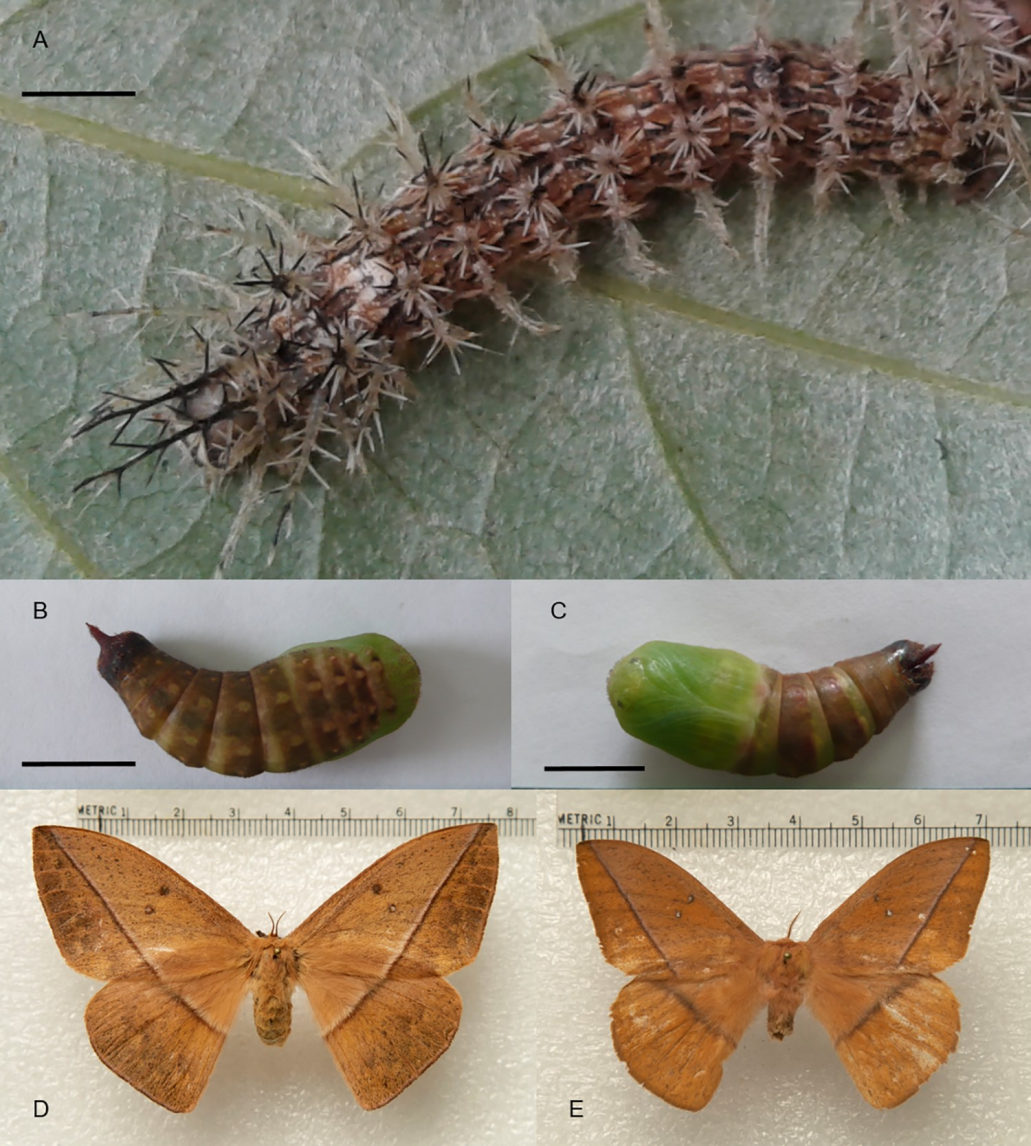
*Lonomia casanarensis* life cycle. (A) Sixth instar larva. (B) Pupae dorsal view. (C) Pupae ventral view. (J) Female. (K) Male. Bar = 1 cm.

Healthy adults were obtained with a sex ratio of 0.55 (44.4% males and 55.5% females) for *L*. *casanarensis*, 0.4 (60% males and 40% females) for *L*. *orientocordillera* and 0.5 (50% males and 50% females) for *L*. *orientoandensis*. Healthy adults of *L*. *columbiana* were not obtained. Females and males generally have similar coloration and patterns (Figs 1I, 1J, 2J, 2K, 3F, 3G, 4D and 4E). Dimorphism could be detected in thinner antennae, larger abdomen, and bigger size and weight in females (Table 1).

*Lonomia orientocordillera* caterpillars suffered attacks by parasitoids (S1 and S2 Figs), which were the main cause of mortality (Table 1). Parasitized caterpillars had a rotten smell, flaccid scoli, evident body weakness, and a smaller size than healthy caterpillars. In addition, some of them had increased abdominal darkening over time. This prevented the movement of their prolegs, making it difficult for them to hold on to plants and ultimately causing them to fall off from the plant. Caterpillars died within two days of beginning to exhibit lethargy. Adult ichneumon wasps emerged 26–28 days after their larvae exited from *L*. *orientocordillera* larvae. *Lonomia casanarensis* parasitoids (S3 Fig) attacked the pupal stage. The parasitized pupa lost weight rapidly and secreted a foul-smelling substance.

### Description of life cycles

Descriptions of the number of colonies and individuals in a colony, total larval growth ratio, total egg to adult mortality, and the parasitoid species and sizes at every stage are reported in Table 1. A more detailed description of the development time, mortality, size, and growth ratio at each stage by instar is shown in Table 2. Data about collection localities and dates, natural environment conditions, and identified host plants are reported in Table 3.

**Table 2:** Detailed description of development time, mortality, size, and growth by stage/instar.

Species	Stage/Instar	Duration of instar (days)	Mean mortality (%)	Mean size (mean cm ± std)	Growth ratio	Cephalic capsule width (mm)
*Lonomia columbiana*	Egg	31 to 35	78	Table 1		
	First instar larvae	8 to 11	0	0.661	^a^	1.074
	Second instar larvae	5 to 6	0	1.002	1.351	1.451
	Third instar larvae	4 to 5	0	1.515	1.391	2.018
	Fourth instar larvae	10	0	2.218	1.386	2.797
	Fifth instar larvae	7 to 11	0	3.147	1.367	3.825
	Sixth instar larvae	13 to 23	0	4.413	1.366	5.226
	Pupa	38 to 45	0	Table 1		
*Lonomia orientocordillera*	Egg	26	37	Table 1		
	First instar larvae	6 to 8	37	0.792	^a^	1.087
	Second instar larvae	7 to 9	1	1.049	1.387	1.507
	Third instar larvae	7 to 10	0	1.319	1.291	1.946
	Fourth instar larvae	8 to 11	0	1.710	1.328	2.585
	Fifth instar larvae	9 to 13	0	2.320	1.384	3.579
	Sixth instar larvae	8 to 19	31	3.458	1.519	5.436
	Pupa	74 to 118	20	Table 1		
*Lonomia orientoandensis*	Fourth instar larvae	7 to?	6	1.638	^a^	2.913
	Fifth instar larvae	7 to 14	18	1.186	1.283	3.737
	Sixth instar larvae	10 to 21	10	5.191	1.420	5.307
	Pupa	98 to 136	31.5	Table 1		
*Lonomia casanarensis*	Fifth instar larvae	9 to?	21	^a^	^a^	3.711
	Sixth instar larvae	7 to 7	38	3.078	1.275	4.731
	Pupa	22 to 129	32	Table 1		

^a^ Data cannot be determined because the life cycle was followed after finding the larvae at a later stage.

**Table 3:** Collection data on four species of *Lonomia* and their identified host plants.

Species	Municipality	Department	Collection Date	Temperature	RH	Altitude (masl)	Host plant	Host plant common name
*Lonomia columbiana*	Pueblo Rico	Risaralda	June 26, 2017	22°C	60%	1421	unknown	unknown
			June 27, 2017			1617	unknown	unknown
*Lonomia orientocordillera*	Cubarral	Meta	July 14, 2017	25°C	75%	694	*Stylogyne sp*. (A. DC.) (Ericales: Primulaceae)	Cucharo
			February 19, 2018			694	*Nephelium cf*. *lappaceum* (Linnaeus) (Sapindales: Sapindaceae)	Rambután
	Leticia	Amazonas	March 14–19, 2018	25°C	70%	90	unknown	unknown
*Lonomia orientoandensis*	Guamal	Meta	June 4, 2018	25°C	75%	unknown	unknown	unknown
*Lonomia casanarensis*	Tauramena	Casanare	November 15–16, 2017	20°C	80%	267	*Schefflera cf*. *morototoni* (Aub*L*.) (Maguire, Steyerm & Frodin) (Apiales: Araliaceae)	Torcazo

### Lonomia columbiana

This species is distributed in Panamá, Costa Rica, Ecuador and along the western mountain range of the Los Andes in Colombia [1]. Of the four species we reared, it exhibits the shortest life cycle, with only 116 to 146 days to complete its development from egg to adult (Table 3). From the 3rd larval instar, the caterpillars display on T3 a conspicuous white to light yellow H-shaped mark (Fig 1C) where the two dorsal lines merge medially; T1-A9 have white to light yellow subdorsal and lateral lines, and thoracic legs and prolegs are light yellow. From the 5th instar, they can be easily recognized by their orange spiracles very contrasting with the greenish-brown bands flanking the body. *Lonomia columbiana* adults have a big size (Table 1). The barcode sequence of *L*. *columbiana* holotype (sample identifier: BC-Her3642) is publicly available in the Barcode of Life Data Systems (BOLD; www.boldsystems.org) within dataset DS-LONO2 ([1]; dx.doi.org/10.5883/DS-LONO2).

### First instar larvae

The description of the first instar larvae is as follows: dark brown cephalic capsule except in the anteclypeus, which is translucent. Yellowish labrum, maxillary palpi, labial palpi and spinneret. Dorsal and ventral sides of the body dark brown. Light brown prolegs. T2 (mesothorax) -T3 (metathorax) with light brown, very thin and weak dorsal lines that converge at the end of T3, forming a conspicuous U. T2 to A1 with a light brown subdorsal line. T1 to T3 have light brown lateral lines. T1-A9 segments with a continuous, thin, light brown subventral line (Fig 1B). Body segments with unbranched scoli, except in T1-T3, which have dorsal and subdorsal scoli bifurcated at the apex and projected above the head, with those of T3 being the longest of the entire body. Scoli in A1-A8 are short and perpendicularly projected from the body axis. A9 with a bifurcated scolus. Dark brown dorsal and subdorsal scoli. Light yellow lateral scoli on the subventral line. T1- A2 and A7- A9 with short, light yellow subventral scoli.

### Second instar larvae

There were no noticeable differences with respect to the previous instar except that larvae were larger, the H-shaped white spot between the T3 and A1 segments of the body and the longitudinal white lines became more conspicuous, and the thoracic spines branched out.

### Third instar

Dark brown cephalic capsule, with darkened labrum and a pale-yellow area between the stemmata and frons. Ventral corners of the frons are dark brown. Dorsal and ventral sides of the caterpillars are brown, with dorsal sides more obscure. T1-A9 with a white dorsal line that joins in T3, forming a conspicuous H spot (Fig 1C). This spot is conspicuous until the sixth stage. T1-A9 have white subdorsal and lateral lines. The space between the dorsal lines forms a dark brown band. The space between the dorsal and subdorsal lines forms a wide dark brown band on which the dorsal scoli are located. The space between the subdorsal and lateral lines forms another dark brown band in which the subdorsal scoli are located. Between the lateral and subventral lines, a lighter brown band is formed in which the spiracles are located. Dark brown dorsal scoli. T3 with branched dorsal scoli longer than those in T1 but shorter than those in T2. A8 with a longer and more robust dorsal scolus than that in A9. Dark subdorsal scoli, shorter than the dorsal scoli and lateral scoli. T1-T3 with branched subdorsal scoli that were longer than in the rest of the body. A9 with the subdorsal scolus as long as the dorsal. Lateral scoli are light yellow in the proximal two-thirds, dark brown in the distal third, and longer than the subventral scoli. Subventral scoli are short and light yellow. Thoracic legs and prolegs are light yellow.

### Fourth instar larvae

Cephalic capsule as in third instar. Subventral area is light brown. T1-A9 with pale yellow dorsal, subdorsal, lateral and subventral lines (Fig 1D). White and conspicuous H spot in T3. The space between the dorsal lines forms a light brown band with dark brown edges. The space between the dorsal and subdorsal lines forms a wide dark brown band on which the dorsal scoli are located. The space between the subdorsal and lateral lines forms another light brown band on which the subdorsal scoli are located. Between the lateral and subventral lines, a dark brown band is formed on which the spiracles are located. Dorsal scoli dark and slightly longer than subdorsal scoli. Subdorsal scoli are shorter than dorsal and lateral scoli and have a dark distal half. T1-T3 with dorsal and subdorsal scoli lengths as in the third instar. A8 has a shorter and more robust dorsal scolus than A9. A9 with scolus projected toward the back. Lateral and subventral scoli as in the third instar. The ventral side, thoracic legs and prolegs of the caterpillar are light yellow.

### Fifth instar larvae

Cephalic capsule as in the third instar, but the dorsal area is light brown, and the anteclypeus is light yellow. Ventral side and subventral area of the caterpillar is light coffee-colored.

T1-A9 with light yellow dorsal and subdorsal lines. T1-A9 with yellow lateral and subventral lines. The space between the dorsal lines forms a light brown band with dark brown edges (Fig 1E). The space between the light yellow dorsal and subdorsal lines forms a wide, dark greenish-brown band on which the dorsal scoli are located. The space between the subdorsal and lateral lines forms a light greenish-brown band on which the subdorsal scoli are located. Between the lateral and subventral lines, a light greenish-brown band is formed on which orange spiracles are located.

The proximal half of the dorsal and subdorsal scoli are light yellow, and the distal half is dark brown. The dorsal scoli are slightly longer than the subdorsal scoli. T1-T3 have dorsal and subdorsal scoli lengths similar to those of the third instar. A8-A9 with lengths and positions of the dorsal and subdorsal scolus as in the fourth instar. Lateral scoli are longer than subdorsal scoli, with the three proximal quarters light yellow and the distal quarter dark brown in color. Subventral scoli as in the third instar, and thoracic legs and prolegs are light yellow.

### Sixth (final) instar larvae

Dark brown cephalic capsule with a light brown dorsal area and a pale-yellow stripe between the stemmata area including frons, although ventral corners of frons are dark brown. The anteclypeus, maxilla, labial palpus and spinnerets are yellowish. The labrum is dark brown.

Ventral side, thoracic legs and prolegs of the caterpillar are pale pink. Subventral area is light brown. T1-A9 with light yellow dorsal, subdorsal, lateral and subventral lines. The space between the dorsal lines forms a light brown band with dark brown edges. Between T2-T3, the dorsal lines are joined, forming a conspicuous H. The space between the light yellow dorsal and subdorsal lines forms a wide, dark greenish-brown band on which the dorsal scoli are located. The space between the subdorsal and lateral lines forms another light greenish-brown band on which the subdorsal scoli are located. Between the lateral and subventral lines, a light greenish-brown band is formed on which the orange spiracles are located (Fig 1F). The dorsal and subdorsal scoli in T1-T3 and A9 are light green on the proximal half and dark brown on the distal half. The central axis of dorsal and subdorsal scoli in A1-A8 are light green, and the setae are reddish brown. The dorsal scoli are slightly longer than the subdorsal scoli, except in T3. T2 with the longest dorsal scoli and T3 with dorsal scoli as short as in A1-A8. A8 with dorsal scolus more robust than the rest of the body. A9 with dorsal and subdorsal scoli shorter than the lateral scoli. The scolus in A9 is projected backward. Lateral scoli are light yellow in the proximal two-thirds and dark brown in the distal third, longer than the subventral scoli. Subventral scoli are short and light yellow, and thoracic legs and prolegs are pale pink.

### Pupa stage

Pupae were dark red right after the caterpillar’s final molt and become dark brown (Fig 1G and 1H) two hours after their formation.

### Adult stage

**Fig 5:**
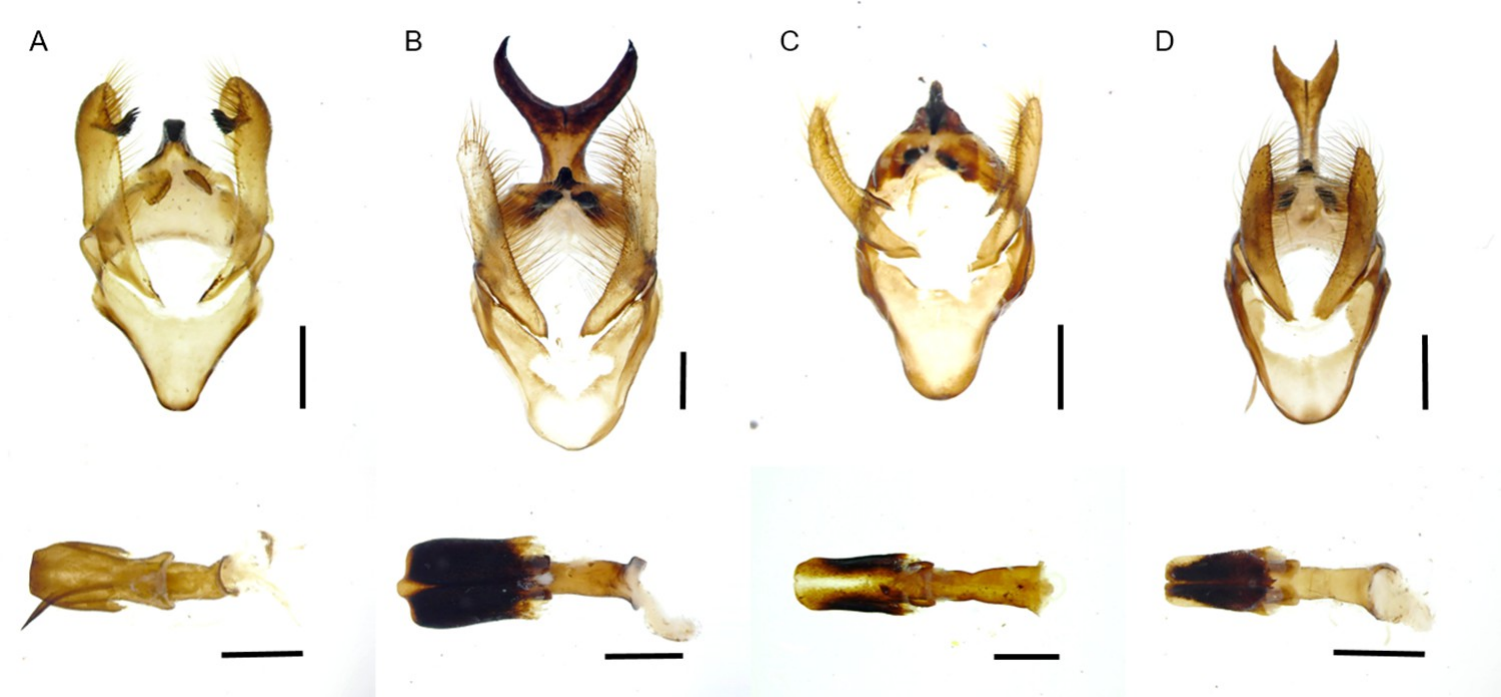
Photographs of male genitalia. (A). *Lonomia columbiana*, (B). *Lonomia orientocordillera*, (C). *Lonomia orientoandensis*, (D). *Lonomia casanarensis*. Bar = 1 mm.

As described by Lemaire [14], the male genitalia have a simple sclerotized uncus (Fig 5A), valves with multiple strong apical and marginal setae, bearing a subapical inner process shaped as a broad sclerotized brush; gnathos forming a pair of arms forming distal flat and rounded lobes with multiple short setae; aedeagus short, slightly curved (ventrally concave) with its vesica bearing a long strongly sclerotized sword-like cornutus. Sclerites of abdominal segment 8 are of regular shape, lacking sclerotized posterior processes found in some other species of the genus.

### Lonomia orientocordillera

This species is distributed in Ecuador, Perú, and the Orinoquía and Amazonas regions of Colombia [1]. Colonies of 6th instar caterpillars were found on *Stylogyne sp* (Primulaceae). and *Nephelium cf*. *lappaceum* (Sapindaceae) host plants. We found that some larvae were parasitized by *Lespesia sp*. (Diptera: Tachinidae) and *Enicospilus sp*. (Hymenoptera: Ichneumonidae). These colonies were reared using healthy leaves of *Prunus serotina* Ehrh (Rosales: Rosaceae) too. The life cycle of *L*. *orientocordillera* is characterized by possessing a life cycle of 145 to 214 days (Table 2). The final larval instars can be easily recognized from the other three species considered here by the dorsal white “V”-shaped mark formed between T3 and A1, and by the light-brown dorsal “V” marks at the posterior ends of A4 and A5 (Fig 2D–2G). The DNA barcode sequence of *L*. *orientocordillera* holotype (sample identifier: BC-HKT 0088) is publicly available in BOLD within dataset DS-LONO2 ([1]; dx.doi.org/10.5883/DS-LONO2).

### First instar larvae

Dark brown cephalic capsule with a long, light-yellow spot on the outer side of the ecdysial lines. Dark brown frons with a light-yellow dorsal corner. Clypeus, anteclypeus, labrum, antennae, maxillary palpi, labial palpi and spinneret are light brown. Dark brown mandibles.

Light brown dorsal body side (Fig 2B). Ventral side, legs and prolegs are pale yellow. T2-A9 with pale yellow dorsal, subdorsal, lateral and subventral lines; The lines do not touch between them. All the scoli are not branched except the dorsal and subdorsal scoli of T1-T3 and the dorsal scoli of A9, which are bifurcated. T1 and T2 scoli are projected above the head; in the rest of the body, the scoli are perpendicular. Dorsal scoli in T2-T3 are the longest. Dorsal scoli of T1 and subdorsal scoli of T1-T3 are a quarter of the length of the T2-T3 dorsal scoli. A1-A9 dorsal scoli measure one-third of the length of the T2-T3 dorsal scoli. The A1-A9 subdorsal scoli are slightly shorter than the subdorsal scoli of T1-T3. T1-A9 lateral scoli are as long as the T1-T3 subdorsal scoli. Subventral scoli are half the size of the laterals. T2-A8 dorsal scoli are dark brown. The dorsal scoli of T1 and A9 and the subdorsal scoli of T1-T3 are pale yellow on the proximal half, and dark brown on the distal half. Subdorsal scoli of A1- A9, lateral and subventral scoli are pale yellow.

### Second instar larvae

Cephalic capsule is dark brown and fades to light brown toward the dorsal side of the epicranial suture. Between the postgenae and over the stemmata areas, a light-yellow stripe is formed, including the frons, although the ventral corners of the frons are dark brown. Space between the stemmata areas to the anteclypeus, and mandibles is dark brown. Dark brown clypeus and mandibles, light yellow anteclypeus and light brown labrum, antennae, maxillary palpi and labial palpi. Dorsal side of the body is light brown (Fig 2C). Ventral side, legs and prolegs are pale yellow. T1-A9 have white dorsal, subdorsal, lateral and subventral lines that do not touch, but widen slightly between T3 and A1 and in A4. Between these white lines, a band of dark brown edges that fades to dark yellow toward the middle is formed.

All scoli are branched. Scoli projection from the body is maintained as in the first instar. T3 have the longest scoli, followed by the dorsal scoli of T2, which are slightly shorter. T1 with dorsal scoli that measure one-third of the T3 size. Dorsal scoli of T1 have one-third of the T3 dorsal scoli. A1-A8 dorsal scoli are as long as T1 dorsal scoli. Dorsal scoli of A9 are slightly shorter than the dorsal scoli of A1-A8. T2 have the longest subdorsal scoli of the whole body, being slightly longer than T1 dorsal scoli. Subdorsal scoli of T1 and T3 are as long as the T1 dorsal scoli. A1-A9 have subdorsal scoli that measure one quarter of the size of the T3 dorsal scoli. Lateral scoli as long as the abdominal subdorsal scoli. Subventral scoli are one quarter of the size of abdominal subdorsal scoli.

The bases of the dorsal and subdorsal scoli are dark brown. The proximal half of the T1 dorsal scoli is pale yellow, and the distal half is dark brown. The proximal quarter of the T2 and T3 dorsal scoli is pale yellow, and the distal three quarters are dark brown. Dorsal scoli of A1-A3 are dark brown. The proximal third of the dorsal scoli in A4-A8 is pale yellow, and the distal two-thirds are dark brown. The three proximal quarters of A9 dorsal scolus are pale yellow, and the distal third is dark brown. The proximal half of the T1-T2 subdorsal scoli is pale yellow, and the distal half is dark brown. In T3-A9, the proximal three quarters of the subdorsal scoli are pale yellow, and the distal quarter is dark brown. Lateral and subventral scoli are pale yellow.

### Third instar larvae

Coloration of the cephalic capsule is similar to that in the second instar. Clypeus, labrum and mandibles are dark brown. Anteclypeus is light yellow, and the antennae, maxillary palpi and labial palpi are light brown. Ventral side and legs are pale yellow. Proleg bases are dark brown. T1-A9 have white dorsal, subdorsal, lateral and subventral lines (Fig 2D). The dorsal and subdorsal lines are wide between T3 and A1 until they join, forming a “V”-shaped white mark. The dorsal lines are widened again at the end of A4 and A5 without joining. Between the white lines, bands of dark brown edges are formed, which fade to dark yellow toward the middle. The projection of the scoli from the body is same as in the first instar.

T2 has the longest dorsal scoli of the body, followed by those in T3, which are slightly shorter. T1 dorsal scoli have half of the T2 scoli dorsal length. Dorsal scoli in A1-A8 are one-third of the dorsal length of the T2 scoli. Dorsal scolus in A8 is as robust as the thoracic dorsal scoli. A9 dorsal scolus as long as in A1-A8, but their branches are shorter and less robust. T2 subdorsal scoli have half of the T2 dorsal scolus length. Subdorsal scoli in T1 and T3 are slightly shorter than the T2 subdorsal scoli. Subdorsal scoli in A1-A9 and all the laterals have half of the A1-A8 dorsal scoli length. Subventral scoli measure one-third of the length of the laterals. The bases of dorsal and subdorsal scoli are dark brown. In T1, the proximal half of the dorsal scoli is yellowish green, and the distal half is dark brown. In T2 and T3, the proximal third of the dorsal scoli is greenish-yellow, and the distal two-thirds are dark brown. In A1-A9, the proximal half of the dorsal scoli is pale yellow, and the distal half is dark brown. In T1-T3, the proximal half of the subdorsal scoli is greenish-yellow, and the distal half is dark brown. In A1-A9, the three proximal quarters of the subdorsal scoli are pale yellow, and the distal quarter is dark brown. Lateral and subventral scoli are pale yellow.

### Fourth instar larvae

Cephalic capsule coloration same as in the second instar but with the light brown gradient of the epicranial suture dorsal side more expanded toward the posterior side. Clypeus, labrum and mandibles are dark brown. Light brown anteclypeus, antennae, maxillary palpi and labial palpi. Coloration of the ventral side, legs, prolegs and lines as in the third instar. Between the white lines, a band of dark brown edges is formed (Fig 2E). The center of the band between the dorsal lines is greenish-yellow, and the other bands retain their dark yellow coloration.

The projection of the scoli from the body is same as in the first instar. T2 dorsal scoli are the longest in the body. Dorsal scoli of T1 and T3 measure half of the length of the T2 dorsal scoli. Dorsal scoli of A1-A8 measure one-third of the length of the T2 dorsal scoli, with the A8 being as robust as the thoracic dorsal scoli. Dorsal scolus of A9 as long as those of A1-A8, but its bristles are shorter and less robust. T2 subdorsal scoli measure half of the length of dorsal scoli in the same segment. The T1 and T3 subdorsal scoli are slightly shorter than those of T2. Subdorsal scoli in A1-A9 have two-thirds of the length of dorsal scoli of the same section. Lateral scoli as long as the dorsal scoli in A1-A9 but thinner. Subventral scoli measure one-third of the length of the lateral scoli. Only the bases of the dorsal scoli are dark brown. Dorsal scoli in T1 and A9 have a greenish-yellow color on the two proximal thirds and a dark brown color on the distal third. Dorsal scoli of T2 have a greenish-yellow color on the proximal third and a dark brown color on the two distal thirds. Dorsal scoli at T3-A8 have a greenish-yellow color on the proximal half and a dark brown color on the distal half. Subdorsal scoli of T1-T3 have a greenish-yellow color on the two proximal thirds and a dark brown color on the distal thirds. A1-A9 with subdorsal scoli that have a pale-yellow color on the three proximal quarters and a dark brown color on the distal quarter. Lateral and subventral scoli are pale yellow.

### Fifth instar larvae

The dark brown color of the cephalic capsule is preserved only near the ventral end of the epicranial suture. It fades to a light brown color in the rest of the capsule. The stripe formed between the postgenae is yellow at this stage. The stemmata area is light brown until they reach the anteclypeus and jaws. Light brown clypeus, labrum, antennae, maxillary palpi and labial palpi. Mandibles are dark brown. Light yellow anteclypeus.

Dorsal side of body is light brown. T1-A9 have white dorsal, subdorsal and lateral lines that are interrupted by the dark brown color from the base of their scoli (Fig 2F). The subventral white line is continuous. The dorsal and subdorsal lines are wide between T3 and A1 until they join, forming a white, “V”-shaped mark. The dorsal lines widen again at the end of A4, where they join, forming a light brown “V”, and again in A5 without joining. Between the white lines, bands with dark brown edges are formed. The color of the bands near the middle is the same as in the fourth stage but is interrupted by the dark coffee-colored bases of the scoli. Ventral side is yellowish pink. Leg coxae and proleg bases are light brown. The projection of the scoli from the body is maintained as in the first instar. T2 dorsal scoli are the longest of body. Dorsal scoli of T1 and subdorsal scoli of T2 measure two-thirds of the dorsal scoli length in T2. The dorsal scoli of T3 measured one-third of those of T2. Dorsal scoli of A1-A8 are slightly shorter than the dorsal scoli of T3. A9 dorsal scolus as long as that of T3 but less robust. Subdorsal scoli of T1 and T3 measure one half of the length of those of T2. Subdorsal scoli of A1- A9 as long as the dorsal scoli of A1-A8 but slightly less robust. Lateral scoli of A1-A8 slightly longer but thinner than their dorsal scoli. Subventral scoli measured one half of the lateral scoli length.

Dorsal scoli in T2 have a light green color on the proximal third and a dark brown color on the two distal thirds. Dorsal and subdorsal scoli in T1, subdorsal scoli in T2 and dorsal and subdorsal scoli in T3- A9 have a light green color on their two proximal thirds and a dark brown distal third. All dorsal and subdorsal scoli bear multiple setae on their distal dark brown part; these setae are basally and distally dark brown, but in their median part are red. Lateral and subventral scoli have light green coloration.

### Sixth (final) instar larvae

The cephalic capsule has a dark brown spot from the ventral end of the epicranial suture that fades to light yellow toward the dorsal side. Under the dark brown spot and between the genae, a white stripe is formed, including the frons, although the ventral corners of the frons are dark brown. Stemmata areas of the anteclypeus and mandibles are dark brown. The clypeus, labrum, antennae, maxillary palp and labial palp are light brown. Anteclypeus is pale yellow.

Ventral side of the caterpillar and prolegs are light brown. Thoracic legs are dark brown. The body coloring pattern is similar to that shown in Fig 2G. The spiracles are dark brown. The lengths and positions of the scoli are similar to those described for *L*. *casanarensis* except that the dorsal scolus in A9 is as long as in T1, the subdorsal scoli in T1 and T3 are as long as the T1 dorsal scoli, the subdorsal scoli in T2 are shorter than the dorsal scoli but slightly longer than the T1 scoli, and the A1-A9 subdorsal scoli are as short as the dorsal scoli in A1-A7 but less robust. Dorsal scoli in T2 have a light green color on the proximal third and a dark brown color on the two distal thirds. Dorsal and subdorsal scoli in T1 and subdorsal scoli in T2 have a light green color on the proximal half and a dark brown color on the distal half. Dorsal and subdorsal scoli in T3-A9 have a light green color on their two proximal thirds and dark brown on their distal third. All the dorsal and subdorsal scoli have identical color to that in the previous instar. T1-A8 have lateral scoli as long as the dorsal scoli of T1, with a light green color on their three proximal quarters and a dark brown color on the distal quarter. Subventral scoli are light green.

### Pupa stage

The newly formed pupae of *L*. *orientocordillera* have an intense light green color on the head and thorax and a light brown color in the abdomen. These colors become darker as the exoskeleton hardens, becoming dark red and then finally dark brown (Fig 2H and 2I).

### Adult stage

Male genitalia present a distinctive uncus armed with a very strong dorso-apical sclerotized bifid process like “bull horns”; valves without inner process, and with multiple apical and marginal setae; gnathos arms converge medially but remain separated, ending as sclerotized lobes with short setae; the juxta is broad, long and strongly sclerotized, tubular in shape; aedeagus short, straight with its vesica bearing a small teeth-shaped cornutus (Fig 5B). The 8^th^ abdominal segment is also distinctive; sternite 8 bears a median pair of large and strong angular teeth on its posterior margin, while tergite 8 ends as a median slightly globulous pointed process (not represented, but see Fig 47 in [24]).

### Lonomia orientoandensis

This species is distributed in Ecuador, Perú, and the Orinoquía region of Colombia [1]. The colonies were found at the transition phase between the 4th and 5th instars. The duration of the entire life cycle remains unknown, but we observe that development from 4^th^ instar larva to adult took 122 to 178 days (Table 2). The final larval instars can be easily recognized by their dark greenish-brown coloration with continuous dorsal, subdorsal and lateral thin white lines, being much more uniform in color that the other species. They present a conspicuous white “V”-shaped mark at the junction between T3 and A1 (Fig 3A–3C); upon closer examination, this mark consists of a pair of pale spots on the posterior margin of T3 (the pale area delimited by the dorsal and subdorsal white lines), and single median pale spot on the anterior margin of A1, between the two dorsal lines. The DNA barcode sequence of the holotype of *L*. *orientoandensis* (sample identifier: BC-RBP 4175) is publicly available in BOLD within dataset DS-LONO2 ([1]; dx.doi.org/10.5883/DS-LONO2).

### Fourth instar larvae

Cephalic capsule with a dark brown spot at the union of the ecdysial lines that fades to light yellow toward the dorsal and lateral sides. Under the dark brown spot and between the postgenae, a light-yellow stripe is formed, including the frons, although the ventral corners of the frons are dark brown. The areas under the light-yellow stripe and between the stemmata to the anteclypeus and mandibles are dark brown. Anteclypeus and clypeus are pale yellow, and the labrum is brown. Antennae, mandibles, maxillary palpi, labial palpi and spinnerets are dark brown.

The dorsal scoli in T2 are the longest in the body. T1 dorsal scoli measure half of the length those of T2. T3 dorsal scoli measure a quarter of the length of those of T2 (Fig 3A). A1-A7 and A9 dorsal scoli are as long as those in T3. Dorsal scoli of A8 are slightly longer and robust than those of A1-A7. Subdorsal scoli of T2 are as long as half of the dorsal scoli in the same segment. The T1 and T3 subdorsal scoli are slightly shorter than those of T2. A1-A8 subdorsal scoli as long as the dorsal scoli but less robust. The A9 subdorsal scoli are slightly shorter and thinner than those of A1-A8. All lateral scoli were slightly longer than the subdorsal scoli. Subventral scoli measure approximately one-third of lateral scoli. T1-T3 dorsal and subdorsal scoli are dark brown with light yellow setae bases and tips. A1-A9 dorsal scoli are dark brown with some light-yellow setae. Lateral and subventral scoli are light yellow.

### Fifth instar larvae

Cephalic capsule same as in the fourth instar but with dark brown dots in light yellow areas and a dark brown clypeus. Ventral side of the caterpillar is yellowish orange. Thoracic leg coxae and proleg bases are light brown. The body is dark greenish-brown with continuous white dorsal, subdorsal and lateral lines (Fig 3B). The subventral line is white but discontinuous, leaving only a white dot between the scoli. The union between the dorsal and subdorsal lines between T3 and A1 forms a white, “V”-shaped mark. The spiracles are dark brown.

Dorsal scoli of T2 are the longest in the body. Dorsal and subdorsal scoli of T1 and subdorsal scoli of T2 are half of the T2 dorsal scoli length. Dorsal scoli of T3 and A9 measured one-third of the T2 dorsal scoli length. Dorsal scoli of A1- A8 are slightly shorter than the T3 dorsal scoli. Subdorsal scoli of T3 are slightly shorter than those of T1-T2. Subdorsal scoli of A1- A8 are as long as the dorsal scoli of the same segments but less robust. In A9, the subdorsal scolus is slightly shorter than in A1-A8. Lateral scoli are slightly longer than the subdorsal scoli in A1-A8. Subventral scoli measure half of the lateral scoli length. The axis of the dorsal and subdorsal scoli is dark brown, but its base and the base of its spines are green. The lateral and subventral scoli are pale yellowish green.

### Sixth (final) instar larvae

Cephalic capsule presents a dark brown spot from the union of ecdysial lines that fades to light yellow toward the dorsal and lateral sides, where there are dark brown points. Under the dark brown spot and between the postgenae, a light-yellow stripe is formed, including the frons, although the ventral corners of the frons are dark brown. The areas underneath the light-yellow stripe and between the stemmata areas to the anteclypeus and the jaws are dark brown. Clypeus, labrum, antennae, maxillary palpi, labial palpi and spinnerets are dark brown. Anteclypeus is pale yellow.

Ventral side of the caterpillar, thoracic legs and prolegs are yellowish orange. Body is dark green with a coloration pattern as shown in Fig 3C. Spiracles are dark brown. The lengths and positions of scoli are similar to those described for *L*. *casanarensis* except that in A1-A7 and A9, the subdorsal scoli are as short as the dorsal scoli of A1-A7 but less robust. In A8, the subdorsal scoli are slightly longer than the rest of the body. Dorsal and subdorsal scoli in T1-T3 are dark brown. The dorsal and subdorsal scoli of A1-A9 are dark brown, but some tips in each scolus are pale green. Lateral and subventral scoli are light yellow.

### Pupa stage

In *L*. *orientoandensis*, the pupae were dark red and later dark brown after a few hours of their formation (Fig 3D and 3E).

### Adult stage

Male genitalia with a simple uncus laterally compressed forming a small sclerotized median crest and with its tip ventral and forming a small sclerotized point; valves simple with multiple apical and marginal setae; the arms of the gnathos form a pair of small apically sclerotized lobes bearing short setae; juxta tubular, strongly sclerotized, except for its ventral surface (Fig 5C). The aedeagus is straight, its vesica with a single very small sclerotized patch with no distinct structure (not represented, see Fig 40 in [25]). Sternite 8 bears, on each side of a median notch of its posterior margin, a pair of sclerotized teeth, the most medial one stronger than the external one (see Fig 40 in [25]); tergite 8 undifferentiated.

### Lonomia casanarensis

This species is distributed in the Orinoquía region of Colombia [1]. We found colonies at the transition phase between the 5th and 6th instars on *Schefflera cf*. *morototoni* (Araliaceae) trees (Table 3). At rearing, we found some pupae parasitized by *Lespesia sp*. larvae. (Diptera: Tachinidae). Development from the 5^th^ larval instar to adult took 38 to 142 days (Table 2). The final larval instars present light-brown body with continuous white and black dorsal, subdorsal and lateral lines, and the white “V”-shaped mark formed by the junction of the dorsal lines between T3 and A1 (Fig 4A). The DNA barcode sequence of *L*. *casanarensis* holotype (sample identifier: BC-RBP 8014) is available in BOLD within dataset DS-LONO2 ([1]; dx.doi.org/10.5883/DS-LONO2).

### Sixth (final) instar larvae

Cephalic capsule with a dark brown spot from the union of ecdysial lines that fades to light yellow toward the dorsal side. A dark yellow stripe is formed under the dark brown spot and between genae, including the frons, although the ventral corners of the frons are dark brown. The area of the stemmata to the anteclypeus and the jaws is dark brown. The clypeus and labrum are dark yellow. Anteclypeus is pale yellow. Antenna, maxillary palp and labial palp and spinnerets are dark yellow.

The ventral side of the caterpillar, thoracic legs and prolegs are light yellow. The coloring pattern is similar to that shown in Fig 4A. The body is light-brown with continuous white with black dorsal, subdorsal and lateral lines. The union between the dorsal and subdorsal lines between T3 and A1 forms a white, “V”-shaped mark. Dorsal and subdorsal scoli in T1-T3 are projected above the head. Dorsal scoli of T1 are long but shorter than those of T2, which are the longest of the whole body. T3 have short and robust dorsal scoli. A1-A7 with dorsal scoli slightly shorter than in T3. The A8 dorsal scoli are as robust as in the rest of the body. Dorsal scoli on A9 are slightly shorter than in T1 and projected toward the posterior side. In T1, the subdorsal scoli have the same length as the dorsal scoli. Subdorsal scoli in T2 are longer than those in the other thoracic segments. A1-A7 and A9 with subdorsal scoli as short as the dorsal scoli of A1-A7 but less robust. In A8, the subdorsal scoli are slightly longer than the rest of the body. Dorsal scoli in T2 have a light-yellow color on the proximal third and a dark brown color on the distal two-thirds. Dorsal and subdorsal scoli of T1 are light yellow. Dorsal scoli of T3-A9 have a light-yellow color on the two proximal thirds and a dark brown color on the distal third. Subdorsal scoli in T2-A8 are light yellow with dark brown tips. Light yellow lateral and subventral scoli.

### Pupa stage

The newly formed pupae of *L*. *casanarensis* have an intense light green color on the head and thorax and a light brown color in the abdomen (Fig 4B and 4C). These colors become darker as the exoskeleton hardens, becoming dark red and then dark brown.

### Adult stage

Male genitalia with the uncus armed with a very long dorsal process bent ventrally and ending as a short bifid fork. The apical tip of the uncus forms a small ventral sclerotized point. Valvae are simple, elongated, with multiple strong apical and marginal setae. Arms of the gnathos form a pair of small apically sclerotized lobes bearing short setae; the juxta is tubular, strongly sclerotized, except for a thin median line on its ventral surface. The aedeagus is straight; vesica lacking cornutus (Fig 5D). The 8^th^ sternite bears a median pair of small pointed spines on its posterior margin; 8^th^ tergite undifferentiated.

## Discussion

Biological and ecological characteristics such as the detailed life cycles, host plants, parasitoids, development time, mortality and growth ratios of four of the fifteen *Lonomia* species reported in Colombia were described in this study. From the collected data on instar durations (Table 2), and the collection dates of the different stages (Table 3), it was possible to estimate the seasonality of the generations of these species. *Lonomia orientocordillera* has two generations per year, one beginning between March and May and another between September and November. Similarly, estimations with colonies of *L*. *casanarensis* and *L*. *orientoandensis* predicted two generations per year beginning in April and October, respectively. *L*. *columbiana* data allowed us to estimate a shorter life cycle of four to five months suggesting there may be as many as three generations per year, starting in February, June, and October.

The egg size of *L*. *columbiana* and *L*. *orientocordillera* was slightly larger than that reported for *L*. *obliqua* and *L*. *electra* (Druce) [21]. The green coloration of the eggs was similar to that reported for *L*. *obliqua* but different from the yellow coloration of *L*. *electra*. The chorion biting behavior has also been observed in *L*. *electra* [21]. The average growth ratio, behavior and scoli distribution were similar among the species, but the scoli length and color patterns of the cephalic capsules, body and scoli differed (see description above for species details). Interestingly, these differences allow for a rather straightforward distinction of these four species, in particular through the consideration of the shape of the T3-A1 dorsal mark (“H” shaped in *L*. *columbiana*, “V” shaped in the other three species), the presence of additional dorsal marks on abdominal segments A4 and A5 (unique to *L*. *orientocordillera*), or coloration patterns (distinctive in *L*. *orientoandensis* and in *L*. *casanarensis*).

The coloration of the newly formed pupa can also be a differentiating character since in *L*. *obliqua*, for example, it is yellowish [19], whereas we found that in *L*. *columbiana* and *L*. *orientoandensis*, it was reddish, and in *L*. *orientocordillera* and *L*. *casanarensis*, it was greenish. We did not identify more important differences than the sizes and color changes with respect to the pupa description of *L*. *obliqua* made by Lorini [16].

Overall, while our results revealed differences in the morphological features of the early stages of the four species studied, we call for caution in their use for species identification, because these stages remain unknown in most of the other 55 or so species [1] within the genus, of which 15 are known to occur in Colombia [1]. It is then unclear if the diagnostic characters discussed above and illustrated in our work would hold further comparisons with other species. It is however important to note that the combination of these proposed characters with information on species distributions (as in [1]) may often narrow down the list of candidate species and make these characters highly relevant for species identification. Unfortunately, the early stages of two other medically important species present in Colombia—namely *L*. *achelous* and *L*. *descimoni*, are either insufficiently known for the former (e.g. in [26]) or undescribed for the later (though its venom was recently studied in [10]). This leaves DNA barcoding as the most reliable method for species identification of *Lonomia* caterpillars at the moment [1], unless these can be reared to adults, thus enabling identification through comparisons of the morphology of male genitalia (e.g. as illustrated for *L*. *descimoni* and *L*. *achelous* in BOLD public data portal (records EL6050 and EL6218 from [1] accessible at http://www.boldsystems.org/index.php/Public_SearchTerms).

In addition to describing the immature stages of four species of *Lonomia* for the first time, we also recorded natural enemies of *L*. *orientocordillera* and *L*. *casanarensis*. As in other Lepidoptera species [27], parasitoid larvae emerged from the integument of their hosts searching for a substrate on which to pupate after exiting the *Lonomia* caterpillar or pupa. We found that *Enicospilus* sp. is parasitoid of *L*. *orientocordillera* larvae. Several species of this genus of Ichneumonidae have been reported as parasitoids of *L*. *obliqua* in Brazil [16,20]. Other studies also reported *Anastatus sp*. (Motschulsky) (Hymenoptera: Eupelmidae) and *Aprostocetus sp*. (Westwood) (Hymenoptera: Eulophidae) as parasitoids of *L*. *obliqua* caterpillars [28]. The emergence of *Lespesia sp*. larvae occurred in the VI instar of the *L*. *orientocordillera* caterpillar, while in *L*. *casanarensis*, it occurred in the pupal stage. The adults of these two parasitoids have different morphologies, suggesting that they belong to different species. *Lespesia* is a genus of the family Tachinidae with greater importance as a parasitoid of *L*. *obliqua* in Brazil [29]. *Lespesia affinis* (Townsend) (Diptera: Tachinidae) has been found to be a parasitoid of *L*. *obliqua* [30]. Considering that these parasitoids are natural enemies of *Lonomia* and the high mortality rate produced in the studied colonies, they may be important control agents of *Lonomia*.

The host plants where we found caterpillars of *L*. *orientocordillera* were *Nephelium cf*. *lappaceum* (Sapindaceae), which is a fruit tree of Asian origin that was recently introduced in Colombia [31], and *Stylogyne sp*. (Primulaceae), which is a neotropical tree used as timber [32]. *Lonomia casanarensis* was found on *Schefflera cf*. *morototoni* (Araliaceae) [33], the most common host plants for *Lonomia* caterpillars in Casanare, which are also neotropical timber trees. However, in this area, five other species of host plants have been reported [12], one of which, *Cananga odorata* (Lam.) Hook. f. & Thomson (Annonaceae), is also exotic. These reports, along with previously available data (*Lonomia* host plants reported as belonging to as many as 29 different families in 14 different orders of plants [34]) and successful captive rearing of caterpillars on *Prunus serotina*, support the assumption that the caterpillars of most *Lonomia* species are polyphagous and rather flexible in the use of available hosts in natural conditions. The Casanare area, one of the areas most affected by encounters with *Lonomia*, has experienced intensification of oil palm plantations, cattle raising, and urbanization, which increases changes in land use, habitat fragmentation, biodiversity loss [12], and changes of host plants, all of which increase the probability of these encounters.

The information generated by this study not only increases our knowledge on the biology and ecology of these species but also provides the opportunity to use this knowledge to generate different prevention strategies. Descriptive information of the traits of the immature stages of these species facilitates the creation of field guides so that they can be easily recognized by rural populations and health departments. The duration of each life cycle stage will make it possible to estimate the months when caterpillars are more likely to be present, and take precautions accordingly. Knowledge of some of the host plants will also shed light on the feeding preferences of these caterpillars and allow farmers to make decisions about planting alternatives for fruit and timber trees. Additionally, extended studies on the parasitoids identified here would open the door to biological control studies. Finally, these results will guide the rearing of immature individuals of other *Lonomia* species in new studies.

## Materials and methods

### Specimen collection

Fieldwork was conducted between June 2017 and June 2018 in five areas: Amazonas, Risaralda, Meta, Casanare, and Guainía (Table 3). A mercury vapor light trap was set up from 18:00 h to 6:00 h to collect adults. Adults that were attracted to the light were captured, and females were kept alive inside paper envelopes until they oviposited. The captured adults were euthanized by injecting ammonium between their metathorax and abdomen. An active search for caterpillars and pupae was conducted by walking throughout vegetation and examining the trunks of medium and large trees where these caterpillars usually perch during the day. In addition, caterpillars were collected in the field when we were notified by local health authorities, mainly the Secretaría de Salud de Tauramena and the Secretaría de Salud del Meta, when rural inhabitants spotted colonies in the field. Live caterpillars were transported in hermetic containers to be reared and identified at the Laboratorio de Ecología Evolutiva y Conservación at the Pontificia Universidad Javeriana-Bogotá. Both adult and larval voucher specimens from each instar were deposited at the Entomology Collection (ANDES-E) center of the Museo de Historia Natural at Universidad de Los Andes.

### Species identification

Species identification was performed using DNA barcodes. In adult specimens, DNA extraction was performed from a foreleg, and to identify immature stages, we used either complete eggs or one caterpillar proleg. Extraction was performed according to the instructions of the ZR Tissue & Insect DNA MiniPrep kit (Zymo Research). The cytochrome c oxidase subunit 1 mitochondrial gene was amplified using the LepF1 (LepF1-ATTCAACCAATCATAAAGATATTGG) and LepR1 (LepR1-TAAACTTCTGGATGTCCAAAAAATCA) primers, obtaining an amplicon of 800 bp.

The PCR was developed in a final volume of 25 μl, containing 12.5 μl of Master Mix Green, 1.25 μl of each primer at 10 μM, 5 μl of nuclease-free water and 5 μl of DNA. The thermal profile consisted of a cycle at 94°C for 1 min, followed by 6 cycles at 94° for 1 min, 95° for 1 min and 30 sec., 72° for a min and 15 sec., followed by 36 cycles at 94° for 1 min, 51° for 1 min and 30 sec., and 72° for 1 min and 15 sec., and a final extension at 72° for 5 min.

The PCR products were revealed on 2% agarose gel. The amplified product was subjected to Sanger sequencing. The obtained electropherograms were edited using the CLC Genomics Workbench 3.6.5 program, and the reverse complement was generated with Gene Runner DNAseq7. Subsequently, these were compared with reference sequences available on BOLD Systems [35] and GenBank (NCBI). The obtained barcode sequences are part of the assembled database DS-LONO2 (dx.doi.org/10.5883/DS-LONO2) published in [1]: CGR_Lon63 for *L*. *columbiana*; CGR_Lon85, CGR_Lon86, CGR_Lon118, and CGR_Lon109 for *L*. *orientocordillera*; CGR_Lon120 and CGR_Lon121 for *L*. *orientoandensis*; and CGR_Lon92, and CGR_Lon93 for *L*. *casanarensis*.

We also dissected male genitalia (*L*. *columbiana* n = 35, *L*. *orientocordillera* n = 14, *L*. *orientoandensis* n = 4, *L*. *casanarensis* n = 8) to match the identification obtained using DNA barcodes with that of the male traits. This was done according to Hardwick [36] by cutting off the abdomen and depositing it in a solution of 10% KOH for 24 hours. After this time, excess KOH was removed by washing the genitalia with alcohol, and the remnant scales of the abdomen integument and sclerites were removed. The abdomen was opened on one side, separating the tergites and sternites to extract the genitalia. The last abdominal sternites and genitalia were preserved in glycerol. Specimen identification was determined by comparing the morphology of the wings and male genitalia with those described by Lemaire [14], Brechlin et al. [25], Brechlin and Meister [24] and Brechlin [37]. Adults were mounted as described by Triplehorn and Johnson [38].

To identify the parasitoids and host plants obtained from the *Lonomia* colonies, tissue samples were sent to Laboratorio Genética de la Conservación in Instituto de Investigación de Recursos Biológicos Alexander von Humboldt for DNA barcoding. The primers used to identify parasitoids were the same used for *Lonomia* identification (LepF1 and LepR1). The primers for ITS regions used to identify host plants were ITS4 (ITS4- TCCTCCGCTTATTGATATCG) and ITS-p5 (CCTTATCAYTTAGAGGAAGGAG).

The photographs that compose the S1, S2 and S3 Figs. were made with LEICA D-LUX 3 digital camera attached to a LEICA M165 C stereomicroscope at different depths standardizing the magnification for each element. The stereomicroscope was equipped with a dome that provided light homogeneously on the structures. Between four and ten photographs were taken at different depths of each element. Each package of photographs was stacked using the Combine ZP program (https://combinezp.software.informer.com) with all the stacking methods.

### Rearing procedures

Eggs laid by females and caterpillars collected in the field were reared to adulthood. They were reared in plastic containers in groups of 10–25 individuals, depending on the number of caterpillars found. Each container was placed in an environmental chamber at the same temperature and relative humidity (RH) conditions of the collection site reported by IDEAM [39] and a 12:12 hour day:night photoperiod to simulate natural conditions. Caterpillars were fed daily with an abundance of fresh leaves of *Prunus serotina* Ehrh (Rosales: Rosaceae). Each rearing container was cleaned daily to avoid proliferation of fungi and bacteria. Once the prepupa phase was reached, a 3 cm layer of clean soil was placed in the container to facilitate pupation. During the pupal phase, the rearing container was kept moist by spraying water on the walls of the container and in the soil. Strips of cloth mesh were hung from the container to facilitate the suspension of adults upon emergence. Adults were sexed and euthanized for later analysis. One caterpillar per instar was euthanized by boiling in water for one minute and then preserved in 99% alcohol. This ensured a relaxed body for later analyses. Adults were euthanized by putting them in a chamber at -20°C for 15 min.

During the rearing process, caterpillars showing coloration changes, bad smells, lethargic behaviors, or liquid secretions were placed into individual containers to avoid colony contamination due to possible bacterial proliferation. Some of the larva and pupae transported to the lab from the field were already parasitized. The parasitized caterpillars were reared under the same conditions as healthy caterpillars to obtain and identify species of parasitoids.

The life cycle descriptions followed the methodology used in other Lepidoptera studies [17,40–42]. For the eggs, we described size and viability. For the larvae, we described the cephalic capsule width, the body length per instar, and the growth ratio that was calculated by dividing the value of the average cephalic capsule width of the molts of one instar by the same data in the previous instar. For the pupae, we described weight and length by sex. For the adults, we described body length, wingspan, and mortality by sex. The full description of the considered traits and the method used to measure or estimate them are given in Table 4.

**Table 4:** Description of the traits measured at each stage or instar and the method used to measure them.

Stage	Trait measured	Method
Egg	Egg size	Average egg height (h), measured on the axis between the area of attachment to the substrate and the opposite end, greater (gD) and smaller (sD) diameters on perpendicular axes to the height
	Duration of egg stage	Number of days counted from the moment of oviposition of the first egg until hatching of the last one^a^
Caterpillar	Larval growth ratio per instar	Average cephalic capsule width of one instar divided by that of the previous caterpillar instar
	Cephalic capsule width	Average distance between the genae
	Body length	Distance between the frons and the last abdominal segment in the relaxed body
	Duration of larval instar	Number of days counted from the moment the first individual molted until the last one molted^a^
Pupae	Weight	Wet body mass of each individual to 0.0001 gr precision.
	Length	Body length by sex from the head to the cremaster tip using a calibrator with a precision of 0.02 mm^b^
	Duration of pupal stage	Number of days counted from the moment the first individual pupated until the last adult emerged^a^
Adults	Body length	Distance between the frons and the last abdominal segment by sex^c^
	Wingspan	Distance in cm between the apices of the forewings
All stages	Egg to adult development time	Number of days from oviposition to adult emergence
	Mortality at each instar/stage	Number of dead individuals divided by the initial number of individuals at each instar/stage

^a^ Because individuals were reared in groups, we were unable to determine the duration of each developmental stage for single individuals.

^b^ Sex was identified according to the characteristics of the VIII and IX abdominal segments described by Lorini and Corseuil [17].

^c^Sex in adults was identified according to the characteristics described by Lemaire [14]: Males are generally smaller and have more vivid colors than females, which are broader, more robust and discreetly colored.

The smallest length measurements were made using a Leica ® S8APO stereomicroscope with an ocular micrometer with a precision of 0.001 mm. Large length measurements were made using a caliper with a precision of 0.02 mm. Weight was measured with a METTLER TOLEDO AB204-S scale with a 0.1 mg precision. Descriptions of caterpillar instars were made using the terminology of Stehr [43]. The calculations of means and standard deviations were made using the version 3.4.0 of R [44].

## Supporting information

S1 Fig Photographs of *Lespesia* sp., parasitoid of *Lonomia orientocordillera*. (A) Lateral view of adult. (B) Larva. (C) Pupa. (D) Cephalic capsule. Bar = 1 mm.
(TIF)

S2 Fig Photographs of *Enicospilus sp*., parasitoid of *Lonomia orientocordillera*. (A) Cephalic capsule. (B) Lateral view of thorax and head. (C) Wings. (D) Larva.
(TIF)

S3 Fig Photographs of *Lespesia sp*., parasitoid of *Lonomia casanarensis*. (A) Lateral view of adult. (B) Cephalic capsule. (C) Wing. Bar = 1 mm.
(TIF)

S1 File Original data obtained from the measurements of the different instars and stages used to describe the *Lonomia* life cycles. (XLSX)
