# Supplementary figures and images for: Characterization of morphological and biological aspects of venomous caterpillars of the genus Lonomia Walker (Lepidoptera: Saturniidae) in Colombia
Source: PLoS One. 2023 May 31;18(5):e0285010. doi: 10.1371/journal.pone.0285010 (PMC10231817; doi:10.1371/journal.pone.0285010)

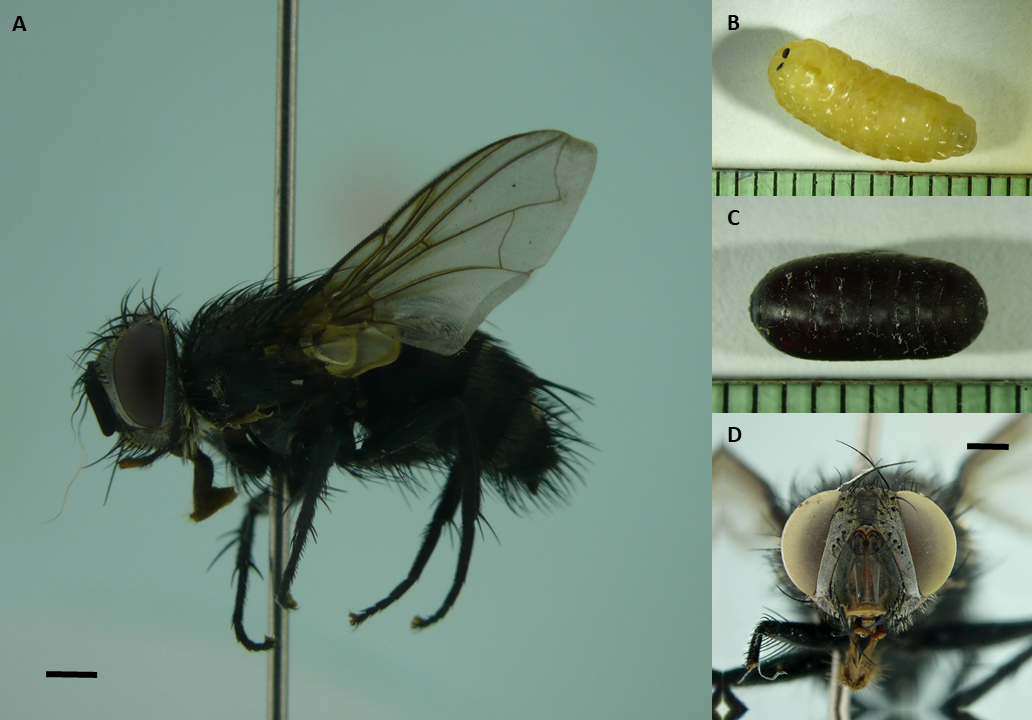

Supplement: S1 Fig — (A) Lateral view of adult. (B) Larva. (C) Pupa. (D) Cephalic capsule. Bar = 1 mm. (TIF) [file pone.0285010.s001.tif]

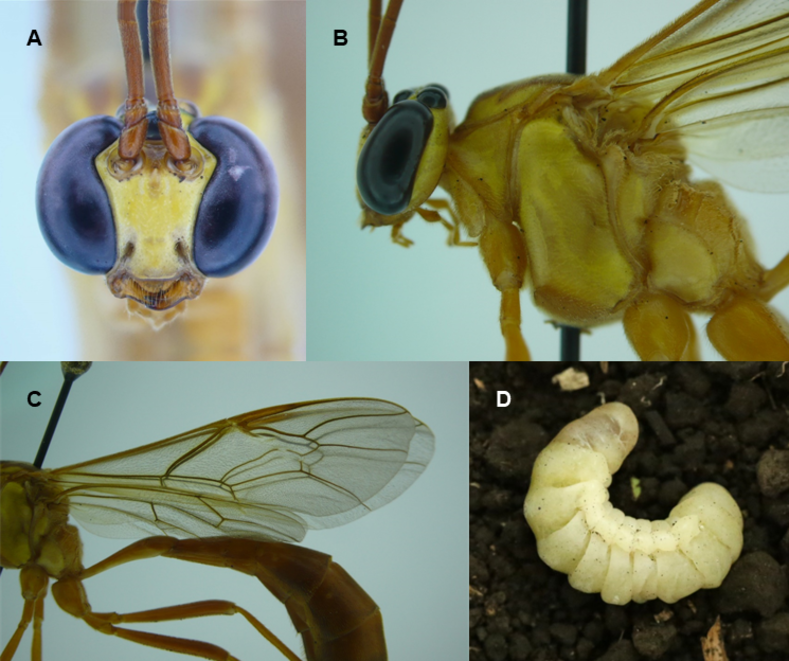

Supplement: S2 Fig — (A) Cephalic capsule. (B) Lateral view of thorax and head. (C) Wings. (D) Larva. (TIF) [file pone.0285010.s002.tif]

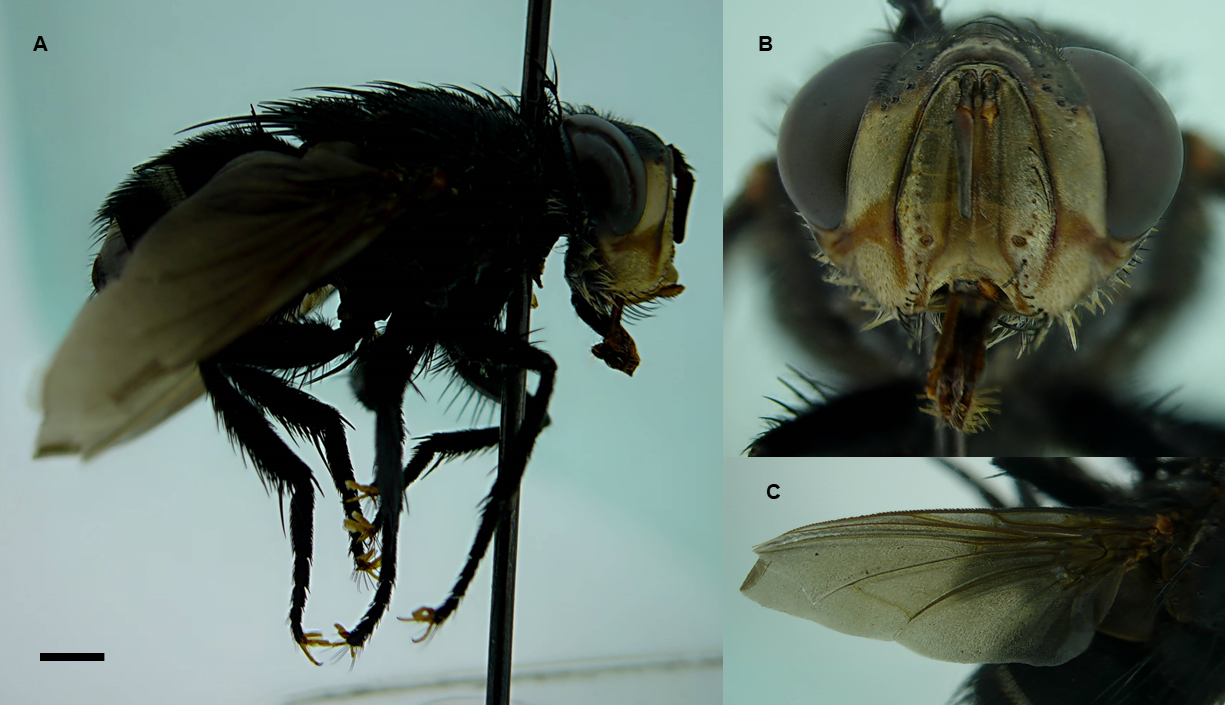

Supplement: S3 Fig — (A) Lateral view of adult. (B) Cephalic capsule. (C) Wing. Bar = 1 mm. (TIF) [file pone.0285010.s003.tif]
